# Exchange rate volatility and manufacturing commodity exports in ASEAN-5: A symmetric and asymmetric approach

**DOI:** 10.1016/j.heliyon.2023.e13067

**Published:** 2023-01-20

**Authors:** Rossanto Dwi Handoyo, Sesotya Putri Alfani, Kabiru Hannafi Ibrahim, Tamat Sarmidi, Tri Haryanto

**Affiliations:** a Department of Economics, Faculty of Economics and Business, Universitas Airlangga, Airlangga Street No. 4, Surabaya 60286, Indonesia; b Department of Economics, Faculty of Social and Management Sciences, Federal University Birnin Kebbi, Kalgo-Bunza Road, 860101, Nigeria; c School of Economics, Faculty of Economics and Management, Universiti Kebangsaan Malaysia, Bangi, Selangor, 43600, Malaysia

**Keywords:** ARCH, ARDL, GARCH, Exports, Manufacturing commodities, Nonlinear-ARDL, Volatility of the exchange rate

## Abstract

This study aims to investigate the influence of the volatility of exchange rates on manufacturing commodity exports in the ASEAN-5 (Indonesia, Singapore, Thailand, Malaysia, and the Philippines). The study used the ARCH/GARCH, ARDL, and Nonlinear ARDL to determine the symmetrical and asymmetrical influence of the volatility of the exchange rate on manufacturing exports in both the short run and long run. Five leading commodity exports for each of the ASEAN-5 countries were used and analyzed over the period January 2007–March 2019. Our strategy using the ARDL approach revealed that volatility has a significant influence on 13 commodity exports in the short term. While the Nonlinear ARDL approach revealed that volatility influenced 19 commodity exports. Additionally, in the long run, finding from ARDL and Nonlinear ARDL also indicates risk-averse behaviour by exporters. However, in the long run, the nonlinear model demonstrates that volatility asserts an asymmetric influence on nearly all commodity exports. With this, therefore, there is the need for policymakers to uphold steadiness in the exchange rate via the use of adequate foreign reserves and amplified the level of investment.

## 1 Introduction

Foreign trade has been known for creating many favourable and unfavourable effects on an open economy's macroeconomic environment. Trade creates absolute and comparative gains long-term prosperity, a stable economic transition, and increase domestic production. Besides, trade has also been known to exacerbate income inequality between and within countries [1,2]. With unexpected fluctuations in the real exchange rates, an open trade policy can negatively affect importers/exporters. This is because such fluctuations affect the current account balance as it reflects changes in the prices of domestic commodities which again will affect the level of output to be exported. Studies by Refs. [3,4] have symmetrically observed the negative effect of volatility on exports in both the short-term and long-term. While studies by Refs. [5,6] noted that when a study uses an asymmetric model, the effect of the volatility of the exchange rate on manufacturing exports is more pronounced. When there exists no differential effect between the negative and positive change in volatility, the effect is said to be symmetrical. While asymmetric volatility occurs when positive and negative changes in volatility are separated or have different effects. This happens because exporters can react differently to a positive and negative change in volatility in which case the influence of volatility on the flow of trade is asymmetrical [7].

The Association of Southeast Asian Nations (ASEAN) is one of the economic organizations that contribute to foreign trade in manufactured goods. In addition, the region has an inflow of foreign direct investment (FDI) that is closely linked to manufacturing exports [8,9]. The inflow of FDI has been increasing from year to year accompanied by increased export performance, especially in the manufacturing sector. Moreover, the exchange rate has been known to affect manufacturing exports. There exists an adverse link between the exchange rate and manufacturing exports in the five ASEAN countries under study. With this nexus, the changes in exchange rate required time adjustment to increase export in the long run so that in the short run the relationship does not match with the theory. There exists a negative link between exports and the volatility in ASEAN-5. While export activities carried out by economic actors are certainly based on risk-averse and risk-takers behaviour due to the risk connected with the volatility of the exchange rate. The risk of uncertainty in the exchange rate occurs because the supply and demand curves of a country's foreign currency can cause the spot and forward rates to change frequently and the exchange rate to fluctuate over time [10]. Risk-averse behaviour is carried out when there is an increase in risk, individuals will avoid risk by reducing their efforts in risky activities and switching to less risky ones, and vice versa for risk takers [11].

With this fact, therefore looking at the exports and the inflow of FDI which are vital factors in an economy, the objective of this study is to analyse the influence of the exchange rate volatility on the main manufacturing commodity exports from ASEAN-5 countries to their main trading partners over the period January 2007 to March 2019. Many previous studies [12,13,14] mainly focused on examining the link between the volatility of exchange rate and aggregate trade flow with little or no regard to disaggregated commodities trade especially commodities at a more disaggregated level (i.e. HS-Code 4 digits level). The only exceptions are [15,16] among others, who analyzed the influence of volatility on HS-Code 2-digit level products. These studies had only focused on HS-Code 2 digits level products while the present study aims to bridge a gap by exploring the volatility of exchange rate effect on HS-Code 4-digit level products that are more disaggregated than the 2-digit products. This is important because most trade policies were mainly focused on aggregate trade, and this study will help provide policymakers with a guide to a more disaggregated commodity trade. However, empirical findings by [17] revealed that the volatility of the exchange rate affects aggregated and disaggregated commodity trade differently. As a result, there is a need to explore the influence of volatility on disaggregated manufacturing exports. With this, therefore, this study is a novel analytical work which explores the asymmetric and symmetric influence of volatility on the export of manufacturing commodities in ASEAN-5. Additionally, while many studies have analyzed the symmetric influence of volatility on export, studies on the asymmetric influence of volatility on export in ASEAN-5 are scarcer to our search for literature. Therefore, this study will furthermore, contribute to the few existing studies that analyzed the asymmetric effect of exchange rate volatility on exports. We add to the literature by considering the exceptional case of ASEAN-5 disaggregated manufacturing exports to the main trading partners. To achieve the stated objective(s) we applied autoregressive distributive lag (ARDL) and nonlinear autoregressive distributive lag (NARDL) model to the collected data of 4-digit manufacturing industrial commodity export (HS-Code 4 digit). Moreover, for a more robustness check and to demonstrate further support to the findings from the ARDL and the NARDL the study also applied quantile regression (at a higher quantile of 90 percentile for the export of manufactured goods.

The need to conduct this study and analyse the impact of exchange rate volatility on manufacturing commodity exports in ASEAN-5 is motivated by the fact that ASEAN-5 countries are emerging and opened to regional and global trade and can be affected by the world economic powerhouse like the United States, Japan, Australia, Hong Kong which featured as their main trading partners. As a result, there is a tendency for possible exchange rate volatility to impact ASEAN-5 trade, especially their exportable commodities. Due to their open trade policy, since 1997 the ASEAN-5 (except for Singapore) were hugely affected by the crisis in the financial market which was necessitated by the highly volatile exchange rates and other macroeconomic indicators. Additionally, the focus on export trade is motivated by the fact that ASEAN-5 countries are export-oriented (although the countries relied heavily on intermediate goods) as their trade is dominated by exports mainly to the nation outside the bloc. Their export has grown significantly over the few decades and their economy is characterized as commodity-based in which export price changes are correlated with exchange rate volatility. However, the focus on ASEAN-5 countries, exports, and the volatility of exchange rate are motivated because volatility's negative impact on export is more pronounced in developing countries than developed world [18].

Using the ARDL method, in this study we found that volatility has a significant influence on 13 commodity exports in the short-term. While the NARDL approach revealed that volatility influenced 19 commodity exports. Additionally, in the long-run, finding from ARDL and NARDL also indicates risk-averse behaviour by exporters. However, in the long run, the NARDL model demonstrates that volatility asserts an asymmetric influence on nearly all commodity exports. These findings, therefore, suggest the need for policymakers to maintain steadiness in the exchange rate via the use of adequate foreign reserves and amplified the level of investment.

Apart from section one which is specifically for the introduction, this study is further divided into four sections which are: part two which elucidates the review of related literature, part three which discusses the methods, data, and data sources, part four which presents the result and discussion, and lastly part five which summarizes the study and offers recommendations for the policymakers.

## 2 Review of literature

A study by [19] was the first to analyse the influence of volatility on trade flow. Their study focuses on exchange rate risk in developed countries in which their findings indicate that the volatility of the exchange rate negatively affects trade. Similarly, in a sample of 13 emerging economies [20], examined the effect of volatility on export, and their findings report that volatility reduces export in both the short run and the long run. This finding has been further supported by Refs. [3,21,22] among other studies. In the short run, a contrary finding by [23] revealed that there is no significant effect of volatility on export while in the long run, there is evidence of a statistically significant effect [24]. correspondingly reports no significant effect of volatility on the trade flow in manufacturing commodities in China, South Africa, and the United States. Reference [25] scrutinized the effect of the volatility of the exchange rate on manufacturing trade in Africa from 1995 to 2012. Their findings indicate that import and export trade are negatively affected by the volatility and real exchange rate in the continent. Reference [26] applied an exponential generalized conditional heteroskedasticity (EGARCH), ARDL, and studied the influence of exchange rate volatility on the exports of manufactured goods from South Africa to the United States from 1990Q1 to 2014Q1. Findings indicate that volatility asserts a significant positive influence on manufacturing export in the long run, and no evidence of a significant effect in the short run. Similarly, in the case of Indonesian exports to the Organization of the Islamic Cooperation (OIC) member states, [15] observed that exchange rate volatility negatively affects the export of some selected commodities export in both the short run and long run.

From 2014 to 2018 [27] observed that volatility asserts a significant negative effect on manufacturing exports of agricultural raw materials, transport, machinery, and equipment and a positive influence on the imports of textiles, fuels, and chemicals products to and from the Russian Federation and its 70 trading partners. In the case of the United States' exports to Brazil, Russia, India, China, and South Africa (BRICS) countries, findings from [28] study revealed that in the long-run exchange rate volatility and the real exchange rate negatively affect exports, while in the short run there exists mixed finding using different estimation techniques. In another study by [29], findings show that the volatility of the exchange rate negatively affects South Africa's export and import to and from the European Union over the period 1980–2009. A contrary finding by [30] reports that the volatility of the exchange rate asserts no significant impact on South Africa's aggregate export [31]. used GARCH and ARDL modelling approaches and scrutinizes the influence of exchange rate volatility on Nigeria's crude oil export to Brazil, Canada, France, Italy, Spain, the USA, and the UK from January 2006 to December 2019. The study results show that the exchange rate volatility significantly affects oil exports from Nigeria to these countries.

Reference [32] used a vector autoregressive model (VAR) and examined the asymmetrical impact of exchange rate volatility on the United States' aggregate exports. The finding indicates that volatility has a negative and significant asymmetric effect on exports. In the case of Malaysian-Thai bilateral trade, [33] observed that the volatility of the exchange rate significantly affects Malaysia's export and import industries. Reference [34] revealed that imports and exports trade respond quickly to fluctuations in price stimuli while their total reactions to time were shorter when the price stimuli are caused by the currency exchange rate.

Reference [35] used the nonlinear ARDL method and observed the asymmetric impact of exchange rates on trade flow between Korea and the United States. Their methodological strategy revealed a substantial impact of exchange rates on trade flows. This has been further supported by studies that used similar approaches such as [18,36].

Studies that investigate the asymmetric effect of exchange rate volatility on trade in some ASEAN-5 countries include [6]; who report that in the short-run exchange rate volatility asserts a significant effect on 32 imported commodities and 22 exported commodities of the Thai industry. On the other hand, the volatility of the exchange rate affects 5 exports and 12 imports commodities in the long run. Another study [7] inspects the influence of volatility on commodity trade between the United States and Malaysia. The study observed asymmetric effects in one-third of Malaysia's export and import sectors. Reference [16] also studied the impact of volatility on the Indonesian primary commodity exported to the United States, South Korea, Japan, India, and China. Finding from nonlinear ARDL revealed that exports from Indonesia to these countries are negatively impacted by the volatility of the exchange rate. Using data from Singapore [37] study confirms that in the short-run the asymmetric impact of the fluctuations in exchange rate occurs in almost all industries. While in the case of the Philippines, volatility has been found to affect exports significantly in Refs. [38,39] studies.

In a nutshell, based on the foregoing literature, the exporters could react differently due to the rise and fall in exchange rate volatility such that the effect of volatility on trade flows can be asymmetrical. As a result, studies concerning the asymmetric impact of the volatility of exchange rates on exports began to develop. The symmetric and asymmetric studies are therefore recommended in the framework of bilateral trade flow based on the type of commodity in the manufacturing industry to prevent bias.

Based on the previous studies, there exists no study that simultaneously observed the symmetric and asymmetric influence of volatility on the export of manufactured goods in ASEAN-5 countries. Therefore, this study aims to bridge this slit in knowledge by simply analyzing the influence of the volatility of the exchange rate on the export of manufactured goods in ASEAN-5 countries. This is important because the share of manufacturing exports remained significant in this sub-region, and among the 10 ASEAN countries only the five ASEAN countries viz; the Philippines, Indonesia, Malaysia, Thailand, and Singapore have available monthly data to allow for the analysis in this study.

## 3 Materials and methods

In this study, autoregressive conditional heteroskedasticity (ARCH) and generalized autoregressive conditional heteroskedasticity (GARCH) were applied to obtain the volatility. This is important because many previous studies used these estimation techniques and estimates the volatility of the exchange rate. According to [40] study as cited in a recent study by [41] ARCH/GARCH is one of the best models to measure the volatility of financial data. Therefore, the ARCH/GARCH estimation technique has been widely used in many recent studies.

However, an ARDL method was also applied to scrutinize the symmetric influence of volatility of the exchange rate on exports, while the nonlinear ARDL method determined the asymmetric effect of volatility on exports. The use of nonlinear ARDL remains suitable in the analysis of the asymmetric influence of the volatility of exchange rate on exports because it offers several advantages. *First*, ECM estimation in one step tends to improve model performance in small samples, especially in terms of cointegration tests. *Second*, it can simultaneously estimate long-term and short-term asymmetry in a simple and easily managed computational way. *Third*, can be applied to both variables in the order of I (0) and the order of I (1) [42].

A monthly time series of secondary data from January 2007 to March 2019 were applied. Moreover, export data at 4-digit HS code commodities were used, industrial production index (IPI), exchange rates in real term, and volatility of exchange rate for ASEAN-5 countries based on leading manufacturing industry commodity exports and their main trading partners were applied. The data for the manufacturing exports comes from the trade map and international trade statistics while the industrial production index and exchange rate were sourced from the international monetary funds (IMF) accessed through the international financial statistics (IFS).

Equation (1) expressed an analytical model for GARCH (1,1) based on [43] as follows:

(1) $$\sigma_{t}^{2}=\gamma_{0}+\gamma_{1}u_{t-1}^{2}+\rho_{1}\omega_{t-1}^{2}$$

In this study, we adopt an analytical model of [16,44], studies, which expressed exports as a function of the index of industrial production of trading partners, exchange rates, and the volatility of the exchange rate. The index of industrial production is applied to proxy the value economic activities of trading partners, this is necessary because the data used is monthly data. The following symmetrical model as expressed in Eq. (2) will be used and processed using the ARDL approach.

(2) $$lnEX_{t}^{i}=a+blnIPI_{t}+clnRER_{t}+dlnVol_{t}+\varepsilon t$$

where; EX is the manufacturing export, IPI is the industrial production index, RER is the exchange rate in real terms, Vol is the fluctuation in the exchange rate measured by its volatility.

Equation (2) depicts the long-run ARDL model estimate. The use of the error correction framework is needed to estimate the short-term effect. By using the ECM model, studies can distinguish short-term effects from long-term effects [45], that is:

(3) $$\Delta \ln\;{EX}_{t}^{i}=\alpha_{0}+\sum_{k=1}^{n1}\alpha_{1}\Delta \ln\;{EX}_{t-k}+\sum_{k=0}^{n2}\alpha_{2}\Delta \ln\;{IPI}_{t-k}+\sum_{k=0}^{n3}\alpha_{3}\Delta \ln\;{RER}_{t-k}+\sum_{k=0}^{n4}\alpha_{4}\Delta \ln\;{Vol}_{t-k}+\beta_{0}\;\ln\;{EX}_{t-1}+\beta_{1}\;\ln\;{IPI}_{t-1}+\beta_{2}\;\ln\;{RER}_{t-1}+\beta_{3}\;\ln\;{Vol}_{t-1}+\varepsilon_{t}$$

In Eq. (3) the variables EX, IPI, and RER, are as defined in Eq. (2), and in addition, Vol denotes the exchange rate volatility. In Eqs. (4) and (5) the study further constructs two new variables, reflecting a positive and negative change in volatility. That is; $Vol_{t}^{+}$ denoting an upsurge in volatility and $Vol_{t}^{-}$, denoting a decrease in volatility. These are expressed in the following form;

(4) $${Vol}^{+}:\sum_{k=1}^{t}\Delta \ln\;{Vol}_{k}^{+}=\sum_{k=1}^{t}\max\left(\Delta lnVolj,0\right)$$

(5) $${Vol}^{-}:\sum_{k=1}^{t}\Delta \ln\;{Vol}_{k}^{-}=\sum_{k=1}^{t}\min\left(\Delta lnVolj,0\right)$$

Therefore, the volatility (Vol) in Eq. (3), is replaced with positive ($Vol_{t}^{+}$) and negative ($Vol_{t}^{-}$) volatility in Eqs. (4) and (5) to obtain an asymmetric model in the form of a nonlinear ARDL method as expressed in Eq. (6):

(6) $$\Delta \ln\;{EX}_{t}^{i}=\theta_{0}+\sum_{k=1}^{n1}\theta_{1}\Delta \ln\;{EX}_{t-k}+\sum_{k=0}^{n2}\theta_{2}\Delta \ln\;{IPI}_{t-k}+\sum_{k=0}^{n3}\theta_{3}\Delta \ln\;{RER}_{t-k}+\sum_{k=0}^{n4}\theta_{4}\Delta \ln\;{Vol}_{t-k}^{+}+\sum_{k=0}^{n5}\theta_{5}\Delta \ln\;{Vol}_{t-k}^{-}+\gamma_{0}\;\ln\;{EX}_{t-1}+\gamma_{1}\;\ln\;{IPI}_{t-1}+\gamma_{2}\;\ln\;{RER}_{t-1}+\gamma_{3}\;\ln\;{Vol}_{t-1}^{+}+\gamma_{4}\;\ln\;{Vol}_{t-1}^{-}+\varepsilon_{t}$$

The study used additional diagnostic checks using the LM test i.e. the Lagrange Multiplier (LM) test of error term serial association, by stating the null hypothesis of “no serial correlation”. Moreover, RESET or Ramsey's test for model misspecification is also used which was established with the hypothesis that there is no misspecification problem in the formulated models. Another test applied is heteroskedasticity using the Breusch-Pagan Godfrey test. Finally, the Wald test is used to examine the asymmetry effect. For more robustness tests, the study used CU and CUQ tests. The CU and CUQ tests are applied to check for model stability using CUSUM and CUSUMQ. Therefore, stable and unstable models are presented as S for “Stable” and U for “Unstable” models.

Furthermore, we used quantile regression in a given dataset to establish a system of the impact of the independent variables on the dependent variable. This is important because quantile regression help to provide a broader look at the nexus between dependent and independent variables. Quantile regression is used in this study because it can provide an additional intuition than the ARDL method in the analysis of the symmetric and asymmetric impact of exchange rate volatility and manufacturing commodity exports in ASEAN-5 countries. So also, quantile regression is used for a reason that the technique can be used in any distribution of the data [46]. With this fact, the method is important in this study because abnormally distributed data are more pronounced in these kinds of analytical studies. Even though our estimates were diagnosed (for normality, homoscedasticity, linearity, and serial correlation) we used quantile regression to provide more robustness checks to the study's findings. We used quantile regression as a measure of robustness because the technique is less likely affected by outliers than the ordinary least square, ARDL, among other methods.

## 4 Results and discussion

### 4.1 Stationary test

In the study, an Augmented Dickey-Fuller (ADF) test for stationarity was applied to all the models i.e. linear ARDL model and nonlinear ARDL model. In Table 1 the findings from the test demonstrate that all the variables in log-transformed are stationary at the level and the first difference and were significant at a 1% level. This suggests that all the variables can be used in the ARDL and NARDL estimation procedures.

**Table 1:** Stationarity test.

	ADF Test	Stationarity Level	Variable	ADF Test	Stationarity Level
Singapore and its trading partners			Thailand and its trading partners		
**a1. Malaysia**			**b1. Australia**		
lnIPI***	−3.925	I (1)	lnIPI***	−3.339	I (1)
lnRER***	−10.972	I (1)	lnRER***	−9.076	I (1)
lnVOL***	−3.592	I (0)	lnVOL***	−11.145	I (1)
lnVOL^−^ ***	−12.608	I (1)	lnVOL^−^ ***	−6.976	I (1)
lnVOL^+^ ***	−11.866	I (1)	lnVOL^+^ ***	−6.420	I (1)
lnEX 8542***	−3.478	I (0)	lnEX 8703***	−14.014	I (1)
lnEX 8517***	−6.855	I (0)	lnEX 8704***	−3.519	I (0)
**a2. United States**			**b2. Japan**		
lnIPI***	−5.198	I (0)	lnIPI***	−4.358	I (0)
lnRER***	−9.353	I (1)	lnRER***	−8.545	I (1)
lnVOL***	−4.971	I (0)	lnVOL**	−3.306	I (0)
lnVOL^−^ ***	−9.723	I (1)	lnVOL^−^ ***	−13.940	I (1)
lnVOL^+^ ***	−8.490	I (1)	lnVOL^+^ ***	−14.038	I (1)
lnEX 8411***	−12.066	I (1)	lnEX 8708***	−2.435	I (1)
lnEX 8486***	−13.460	I (1)	lnEX 8542***	−3.591	I (0)
**a3. Japan**			**b3. Malaysia**		
lnIPI***	−4.358	I (0)	lnIPI***	−3.925	I (1)
lnRER***	−9.694	I (1)	lnRER***	−10.475	I (1)
lnVOL***	−9.068	I (0)	lnVOL***	−6.441	I (0)
lnVOL^−^ ***	−3.716	I (0)	lnVOL^+^ ***	−13.871	I (1)
lnVOL^+^ ***	−2.984	I (0)	lnVOL^+^ ***	−13.807	I (1)
lnEX 8471***	−11.868	I (1)	lnEX 8471***	−9.118	I (1)
**Malaysia and its trading partners**			**Indonesia and its trading partners**		
**c1. Hongkong**			**d1. Malaysia**		
lnIPI***	−4.935	I (0)	lnIPI***	−3.925	I (1)
lnRER***	−9.180	I (1)	lnRER***	−9.789	I (1)
lnVOL***	−4.066	I (0)	lnVOL***	−3.840	I (0)
lnVOL^−^ ***	−9.326	I (1)	lnVOL^−^ ***	−4.609	I (1)
lnVOL^+^ ***	−10.456	I (1)	lnVOL^+^ ***	−12.674	I (1)
lnEX 8542***	−13.660	I (1)	lnEX 7219**	−3.080	I (0)
lnEX 8523***	−11.297	I (1)	lnEX 3823**	−3.190	I (0)
**c2. United States**			**d2. Philippines**		
lnIPI***	−5.198	I (0)	lnIPI***	−3.225	I (1)
lnRER***	−8.939	I (1)	lnRER***	−9.662	I (1)
lnVOL***	−6.647	I (0)	lnVOL***	−4.208	I (0)
lnVOL^−^ ***	−10.115	I (1)	lnVOL^−^ ***	−12.430	I (1)
lnVOL^+^ ***	−10.624	I (1)	lnVOL^+^ ***	−12.591	I (1)
lnEX 8541***	−11.483	I (1)	lnEX 8703***	−10.432	I (1)
lnEX 4015***	−14.314	I (1)			
**c3. Singapore**			**d3. United States**		
lnIPI***	−16.184	I (1)	lnIPI***	−5.198	I (0)
lnRER***	−10.972	I (1)	lnRER***	−9.434	I (1)
lnVOL**	−3.449	I (0)	lnVOL***	−17.554	I (0)
lnVOL^−^ ***	−5.863	I (1)	lnVOL^−^ ***	−18.382	I (1)
lnVOL^+^ ***	−11.494	I (1)	lnVOL^+^ ***	−12.299	I (1)
lnEX 8471***	−12.860	I (1)	lnEX 6403**	−11.245	I (1)
**Philippines and its trading partners**					
**e1. United States**			**d4. Japan**		
lnIPI***	−5.198	I (0)	lnIPI***	−4.358	I (0)
lnRER***	−9.032	I (1)	lnRER***	−9.160	I (1)
lnVOL***	−3.558	I (1)	lnVOL***	−5.882	I (0)
lnVOL^−^ **	−3.236	I (0)	lnVOL^−^ ***	−12.447	I (1)
lnVOL^+^ ***	−3.574	I (1)	lnVOL^+^ ***	−13.768	I (1)
lnEX 8542***	−15.724	I (1)	lnEX 4802**	−2.898	I (0)
lnEX 8471***	−15.974	I (1)			
lnEX 8543***	−14.654	I (1)			
lnEX 8544***	−3.630	I (0)			
lnEX 8443***	−13.411	I (1)			

Source: Authors' processed data.

*** symbolizes a 1% significance level, ** symbolizes a 5% significance level, and * symbolizes a 10% significance level.

### 4.2 Cointegration test

A cointegration test was conducted to detect the long-term nexus between the variables. A model is said to be cointegrated in the long term if the value of the F-statistic is greater than the upper bound. On the other hand, a model is not cointegrated if the value of the F-statistic is below the lower bound. The ARDL upper-bound values at a significance level of 1%, 5%, and 10% are given by 5.61, 4.35, and 3.77 respectively. Meanwhile, the lower bound value of ARDL at the significance level of 1%, 5%, and 10% are given at 4.29, 3.23, and 2.72, respectively. Table 2 displays the findings from the cointegration test for the ARDL and NARDL models. The findings from the ARDL model show that 10 commodities are cointegrated in the long run. In the long run, the commodities that are cointegrated include; commodity codes 8517 and 8471 in Singapore; 8708, 8542, and 8704 in Thailand; 8523 in Malaysia; 8703 in Indonesia; 8542, 8471, and 8544 in the Philippines. These commodities were cointegrated at different levels of significance.

**Table 2:** Cointegration test.

Code	F-stat.		Code	F-stat.	
	ARDL	Nonlinear ARDL		ARDL	Nonlinear ARDL
Singapore			Indonesia		
8542	2.542	3.287	8703	6.351***	6.952***
8411	1.863	4.266**	7219	2.131	2.096
8517	11.280***	8.735***	3823	1.957	5.041**
8486	1.047	2.568	6403	1.161	13.277***
8471	5.104**	30.942***	4802	3.274	4.891**
**Thailand**			**Philippines**		
8471	1.183	1.924	8542	5.480**	6.273***
8703	3.435	2.154	8471	5.172**	7.214***
8708	4.170*	3.692*	8543	2.541	2.690
8542	6.675***	4.942**	8544	9.707***	6.474***
8704	7.168***	7.652***	8443	2.828	3.738*
**Malaysia**					
8542	1.528	2.900			
8541	1.127	4.352**			
8471	1.819	3.378			
8523	3.773*	2.247613			
4015	1.728	3.015			

Source: Authors' processed data.

*** symbolizes a 1% significance level, ** symbolizes a 5% significance level, and * symbolizes a 10% significance level.

### 4.3 ARDL test

After obtaining the value of the volatility of the exchange rate from the selected ARCH/GARCH model, the ARDL test is carried out to examine the symmetrical impact of the volatility of the exchange rate. Table 3 reports the result of short-run ARDL estimation for conditional volatility. The findings demonstrate that volatility has a short-term significant positive impact on electronic integrated circuits (8542) and Turbojets and turbo propellers (8411) in Singapore, automatic data-processing machines (8471), parts and accessories for tractors, motor vehicles (8708), and electronic integrated circuits (8542) in Thailand, automatic data-processing machines (8471) in Malaysia, footwear with outer soles of rubber, plastics, leather (6403) in Indonesia, insulated, wire, cable (8544) in the Philippines. These findings imply that in the short run and contrary to our expectation high volatility of exchange rate increases export and profitability of exporters. The positive effect also tells that exporters are risk-takers as they tend to trade more despite the high volatility of the exchange rate. Meanwhile, volatility asserts a significant negative impact on telephone sets (8517) in Singapore, motor vehicles for the transport of goods (8704) in Thailand, motor cars and other motor vehicles (8542) in Malaysia, and Flat-rolled products of stainless steel (7219) in Indonesia. The negative influence explained that in ASEAN-5 countries, high volatility of exchange rate reduces export and profits of exporters for commodities such as telephone sets, motor vehicles for transport, motor cars and vehicles, and products of stainless steel respectively. In addition, the volatility also asserts a negative and positive effect at different lags in the Philippines (8542). This finding provides a mixed impact of exchange rate volatility on electronic integrated circuits commodity. This indicates that exporters of electronic integrated circuits are neither risk-takers nor risk-averse in the short run.

**Table 3:** Short-term ARDL estimation (conditional volatility).

Code	Commodity	Short-term				
		ΔlnVol_t_	ΔlnVol_t-1_	ΔlnVol_t-2_	ΔlnVol_t-3_	ΔlnVol_t-4_
Singapore						
8542	Elect. integrated circuits	−0.002	0.012	–	0.065***	–
8411	Turbojets, turbo propellers	0.018	0.040	0.088**	0.119***	–
8517	Telephone sets	−0.003	−0.143**	−0.085	−0.188***	–
8486	Machines and apparatus	–	–	–	–	–
8471	Data-processing machines	–	–	–	–	–
**Thailand**						
8471	Data-processing machines	0.034**	–	–	0.065***	–
8703	Motorcars and vehicles	–	–	–	–	–
8708	Parts for tractors, motor vehicles	−0.003	0.068**	–	–	–
8542	Elect. integrated circuits	−0.022	0.067	0.036	0.150***	0.114***
8704	Motor vehicles for transport.	−0.128***	–	–	–	–
**Malaysia**						
8542	Motorcars and vehicles	−0.183**	−0.139*	–	–	–
8541	Diodes, transistors & devices	–	–	–	–	–
8471	Data-processing machines	0.011	0.069	0.163**		
8523	Discs, tapes, solid, and storage devices	–	–	–	–	–
4015	Apparel & Clothing	–	–	–	–	–
**Indonesia**						
8703	Motorcars and vehicles	–	–	–	–	–
7219	Products of stainless steel	0.023	0.350	0.199	−0.640**	–
3823	Industrial monocarboxylic acids	–	–	–	–	–
6403	Footwear/plastics/leather	0.013	0.081***	0.050**	–	–
4802	Paper & paperboard	–	–	–	–	–
**Philippines**						
8542	Elect. integrated circuits	−8.804***	8.699***	–	–	–
8471	Data-processing machines	–	–	–	–	–
8543	Electrical mach. & Apparatus	–	–	–	–	–
8544	Insulated, wire, cable	−1.149	3.652	−8.773	6.966*	–
8443	Printing machinery	–	–	–	–	–

Source: Authors' processed data.

*** symbolizes a 1% significance level, ** symbolizes a 5% significance level, and * symbolizes a 10% significance level.

Table 4 displays that in the long-term, in the case of Singapore, there is no evidence of significant impact (either positive or negative) of volatility on the analyzed commodities. This result implies that Singapore has a strong policy that will convert the expected harmful impact of volatility on export in the long run. The volatility of the exchange rate asserts a positive and significant influence on the export of automatic data-processing machines (8471) in the Philippines. This implies that despite high volatility in the exchange rate in the long run exporters are risk-takers and tend to export more automatic data-processing machines and earn high profits. A significant negative influence is observed on motor cars and other motor vehicles (8703), motor vehicles for the transport of goods (8704), parts and accessories for tractors, motor vehicles (8708), electronic integrated circuits (8542) in Thailand, discs, tapes, solid-state non-volatile storage devices (8523) in Malaysia, uncoated paper and paperboard (4802), motor cars and other motor vehicles (8703) in Indonesia, electronic integrated circuits (8542), insulated, wire, cable (8544) in the Philippines. This means that exporters of these commodities are risk averse as they tend to reduce export during periods of the high volatility of exchange rates. Since volatility is characterized by uncertainty, to mitigate the negative effect of volatility on export there is a need to consider the use of hedging in the exchange rate. Meanwhile, in the long-term, the exchange rate asserts a positive and significant impact on telephone (8517) sets in Singapore, electronic integrated circuits (8542) in Thailand, discs, tapes, solid-state non-volatile storage devices in Malaysia (8523), uncoated paper and paperboard (4802) in Indonesia, and automatic data-processing machines (8471), electrical machines and apparatus (8543), insulated, wire, cable (8544) in Philippines. While a negative and significant impact exists on automatic data-processing machines (8471) in Singapore. This result indicates that the depreciation of the Singaporean dollar against the currency of export destination countries will reduce export. This finding is not consistent with our theoretical a priori. The exchange rate asserts a negative effect on export because changes in the exchange rate are incorporated and relative to the changes in the price level. The trading partners’ industrial production index (IPI) positively and significantly impact Singapore (8517), Indonesia (8703, 7219), and the Philippines (8443). These findings are consistent with our expectations and are supported by Refs. [16,47,48] that an improvement in production or economic activities in the ASEAN-5 will increase exports. In the same vein decrease in economic activities in destination, countries could result in a decrease in demand and export for ASEAN-5 countries. While IPI asserts a negative significant impact on Thailand (8704), Malaysia (8523), and the Philippines (8542) in the long run. This is contrary to the theoretical a priori and implies that the export of these commodities decreases with an improvement in economic activities.

**Table 4:** Long term ARDL estimation (conditional).

Code	C	lnIPI	lnRER	lnVol
Singapore				
8542	1.521**	0.738	1.340	−0.123
8411	−2.228	8.872	6.593	−2.661
8517	1.421	2.650***	2.350**	0.172
8486	2.682	−3.902	−6.648	−0,183
8471	1.632	0.537	−0.845***	−0.132
**Thailand**				
8471	1.749	−7.252	−22.562	0.126
8703	6.982	−1.963	−2.075	−0.453*
8708	1.369	−0.005	0.745	−0.482***
8542	3.370**	−1.192	2.469***	−0.524**
8704	9.281**	−4.688*	1.533	−0.683***
**Malaysia**				
8542	2.660	−1.854	1.429	−0.426
8541	2.595	−18.929	4.082	−0.377
8471	0.279	0.741	2.189	−0.500
8523	10.373*	−18.129*	8.603***	−2.495**
4015	1.248	−0.833	0.913	−0.040
**Indonesia**				
8703	−3.979*	4.215***	0.629	−0.132**
7219	−17.432	6.819*	5.005	0.261
3823	0.877	4.902	−1.960	−0.477
6403	0.379	−3.080	2.715	−0.327
4802	0.737	0.004	1.668**	−0.179*
**Philippines**				
8542	15.515***	−11.228***	−0.076	−1.430***
8471	−1.950	1.368	3.140***	0.514***
8543	−52.209***	23.084	37.544***	0.119
8544	3.437*	−1.408	2.018***	−0.545***
8443	−82.543***	53.827***	−0,679	−1.207

Source: Authors' processed data.

*** symbolizes a 1% significance level, ** symbolizes a 5% significance level, and * symbolizes a 10% significance level.

The study also tests for unconditional volatility. The purpose of the test is to verify the reliability of the findings and to test the robustness of the model with the same variables but different measurements. Table 4 shows that there are only 8 out of 25 commodities whose results are inconsistent in the long term. This indicates that most estimated models are robust and consistent with ARDL test results in the short term.

On the other hand, in Tables 5 and 6 we test for unconditional volatility using the ARDL method in the short term and also show the same results as in the long term, that is, 7 out of 25 commodities have inconsistent results (Table 5). Therefore, it can be indicated that most of the models are robust and consistent with ARDL test results in the short term.

**Table 5:** Long term ARDL estimation (unconditional).

Code	C	lnIPI	lnRER	lnVol
Singapore				
8542	–	0.796	1.078	−0.101
8411	–	1.467*	18.34509	−2.506
8517	–	2.169**	2.048*	0.244*
8486	–	−0.685	−5.076***	0.402
8471	–	0.609	−0.781***	−0.071
**Thailand**				
8471	–	0.071	−0.881	−0.016
8703	–	−2.798	−3.431**	−0.451*
8708	–	3.325	3.237	−0.103
8542	–	−2.350*	1.036**	−0.202*
8704	–	−4.842*	0.335	−0.428**
**Malaysia**				
8542	–	2.698***	3.242***	−0.717**
8541	–	0.844	−2.186	1.127
8471	–	−2.107	4.069***	0.232
8523	–	0.101	8.315**	−0.059
4015	–	1.073	1.178	−0.177
**Indonesia**				
8703	–	3.947***	−1.355	−0.203
7219	–	8.559**	4.92	−0.306
3823	–	4.217	−5.866	0.218
6403	–	2.800	−0.634	0.400
4802	–	−1.493	1.669**	−0.307*
**Philippines**				
8542	–	−1.516	−1.075	−0.68**
8471	–	−2.107	4.069***	0.232
8543	–	29.936**	37.921***	−0.524
8544	–	−0.276	1.948**	−0.648***
8443	–	50.912***	1.102	−1.393

Source: Authors' processed data.

*** symbolizes a 1% significance level, ** symbolizes a 5% significance level, and * symbolizes a 10% significance level.

**Table 6:** Short-term ARDL estimation (unconditional).

	Commodity	Short Term										
		ΔlnVol_t_	ΔlnVol_t-1_	ΔlnVol_t-2_	ΔlnVol_t-3_	ΔlnVol_t-4_	ΔlnVol_t-5_	ΔlnVol_t-6_	ΔlnVol_t-7_	ΔlnVol_t-8_	ΔlnVol_t-9_	ΔlnVol_t-10_
Singapore												
8542	Elect. integrated circuits	–	–	–	–	–	–	–	–	–	–	–
8411	Turbojets & propellers	0.530**	–	–	–	–	–	–	–	–	–	–
8517	Telephone sets	–	–	–	–	–	–	–	–	–	–	–
8486	Machines and apparatus	–	–	–	–	–	–	–	–	–	–	–
8471	Auto. data-pro mach.	–	–	–	–	–	–	–	–	–	–	–
**Thailand**												
8471	Auto. data-pro mach.	–	–	–	–	–	–	–	–	–	–	–
8703	Motorcars and vehicles	–	–	–	–	–	–	–	–	–	–	–
8708	Parts & acc for tractors	−0.105*	–	–	–	–	–	–	–	–	–	–
8542	Elect. integrated circuits	0.025	−0.228**	–	–	–	–	–	–	–	–	–
8704	Motor vehicles for transport.	0.207	0.252	0.333	0.278	−0.891**	1.081***	−0.352	0.294	−0.186	−0.582	0.920***
**Malaysia**												
8542	Motorcars and vehicles	−0.017	0.075	0.216	0.274**	–	–	–	–	–	–	–
8541	Diodes, transistors & dev.	−0.142	–	–	–	–	–	–	–	–	–	–
8471	Auto. data-pro mach.	–	–	–	–	–	–	–	–	–	–	–
8523	Discs, tapes, & other dev.	–	–	–	–	–	–	–	–	–	–	–
4015	Apparel & Clothing	–	–	–	–	–	–	–	–	–	–	–
**Indonesia**												
8703	Motorcars and vehicles	–	–	–	–	–	–	–	–	–	–	–
7219	Products of stainless steel	–	–	–	–	–	–	–	–	–	–	–
3823	Monocarboxylic acids	–	–	–	–	–	–	–	–	–	–	–
6403	Footwear/plastics/leather	–	0.189**	−0.156*	–	–	–	–	–	–	–	–
4802	Paper & paperboard	–	–	–	–	–	–	–	–	–	–	–
**Philippines**												
8542	Elect. integrated circuits	−0.318*	0,099	0.072	0.401**	–	–	–	–	–	–	–
8471	Auto. data-pro mach.	–	–	–	–	–	–	–	–	–	–	–
8543	Elect. Mach. & Apparatus	–	–	–	–	–	–	–	–	–	–	–
8544	Insulated, wire, cable	−0.184	0.252*	0.014	0.264*	−0.197	0.443***	−0.375***	0.418***	–	–	–
8443	Printing machinery	–	–	–	–	–	–	–	–	–	–	–

Source: Authors' processed data.

*** symbolizes a 1% significance level, ** symbolizes a 5% significance level, and * symbolizes a 10% significance level.

The ARDL result which shows the symmetrical effect of the volatility of the exchange rate states that the volatility asserts a significant impact on thirteen commodities in ASEAN-5 in the short term. Meanwhile, the nonlinear ARDL reports that 19 commodities are significantly affected by the volatility of the exchange rate. The findings are consistent with [6] who observed that there is a rise in the number of industries affected by the volatility of the exchange rate in a nonlinear model.

In the short term, volatility asserts a positive and negative influence on exports. *Firstly*, volatility has a negative impact on exports. Table 3 results indicate that a 1% rise in volatility will result in Singapore's exports of commodity 8517 (Telephone sets) to decrease by 0.14% at the first lag and 0.18% at the third lag. These findings are consistent with [3] who observed that volatility asserts a negative influence on exports. This adverse influence simply connotes that exporters are risk-averse. *Secondly*, volatility has a positive impact on exports. This finding illustrates that an increase in Singapore's volatility of exchange rate by 1%, will be accompanied by a 0.12% increase in exports of commodity 8411 (Turbojets, turbo propellers). This positive effect suggests that with the rising volatility of the exchange rate exporters are risk-takers. While from Table 4, in the long run, volatility asserts a negative and significant impact on Thailand (8703, 8704, 8708, 8542), Malaysia (8523), Indonesia (8703, 4802), and a significant positive effect on the Philippines (8471). This suggests that a 1% rise in the volatility of the exchange rate in Thailand will be accompanied by a 0.45% decrease in 8703 (Motor cars and other motor vehicles) commodity export. However, a 1% increase in exchange rate volatility will increase 8471 (automatic data-processing machines) commodity export in the Philippines.

The ARDL diagnostic test results in Appendix A show that in all the estimated models the coefficient of ECM_t-1_ is significant and negative at the 1% and 5% levels. Therefore, the value of ECM_t-1_ can be said to be valid. The value of the ECM_t-1_ coefficient describes the adjustment process toward equilibrium in the long run. The largest ECM_t-1_ coefficient (i.e. −0.579) was found in Singapore for the commodity (8471). This means that the adjustment process to long-term equilibrium is 1.7 months (i.e. 1/0.579). The largest ECM_t-1_ coefficient (i.e. 0.337) in Thailand appeared in commodity (8704). This suggests that the adjustment process to long-term equilibrium is 3 months (i.e. 1/0.377). Furthermore, the largest ECM_t-1_ coefficient (i.e. 0.143) in Malaysia is found in commodities (8703) and (8471). This indicates that the adjustment process to long-term equilibrium is 7 months (i.e. 1/0.143). In Indonesia, the largest ECM_t-1_ coefficient (i.e. −0.307) is found in commodity (8703) with the speed of adjustment to the long-term equilibrium of 3.3 months (i.e. 1/0.307). While in the Philippines the largest ECM_t-1_ coefficient (i.e. 0.340) appeared in commodity (8443) with the speed of adjustment to the long-term equilibrium of 2.9 months (1/0.340).

The Lagrange Multiplier test shows the absence of autocorrelation in almost all models, this is because the tests indicate a probability value of more than 5% in almost all models. If the resulting probability exceeds the 5% significance level, then the model is free from autocorrelation problems. Likewise, the Breusch Pagan Heteroscedasticity test shows that almost all models do not show signs of heteroscedasticity. Adjusted R^2^ in almost all the models explained more than 50% of variations in the dependent variables. While findings from the Ramsey RESET test show that almost all models are well-specified. This is because the results show the test's probability value of more than 5%.

### 4.4 NARDL test

We used NARDL to compare and see whether the effect of exchange rate volatility on export follows a similar pattern as in the case of ARDL. Therefore, in this case, we test for the asymmetric influence of exchange rate volatility on export in which exporters could respond to positive or negative changes in volatility. Findings in Table 7 show that an increase in volatility positively affects the export of electronic integrated circuits (8542) and negatively affects the export of telephone sets (8517) and Machines/apparatus for the manufacture of semiconductors (8486) in Singapore. While at different lags a positive change in volatility asserts both positive and negative effects on turbojets, turbo propellers (8411) and (8471) automatic data-processing machines in Singapore. This implies that an increase in volatility promotes the export of (8542) and harms the export of (8517) and (8486) while at the same time promoting and harming Singaporean exports of (8411) and (8471) commodities at different lags. This result further suggests that there is a mixed effect of an increase in volatility on certain commodities exports in Singapore. The negative change in volatility promotes the export of telephone sets (8517) and harms the export of automatic data-processing machines (8471) in the short run. In the long run, as depicted in Table 8 our finding only suggests a positive effect of both an increase and decrease in volatility on automatic data-processing machines (8471) commodity export in Singapore. These findings are consistent with the symmetric result which shows a significant effect of volatility in both the short run and long run. The degree to which volatility affects export is higher for a positive change in volatility than for a negative change in volatility.

**Table 7:** Short-term NARDL estimate.

Code	Short Term											
	ΔlnVol^+^_t_	ΔlnVol^+^_t-1_	ΔlnVol^+^_t-2_	ΔlnVol^+^_t-3_	ΔlnVol^+^_t-4_	ΔlnVol^+^_t−5_	ΔlnVol^−^_t_	ΔlnVol^−^_t−1_	ΔlnVol^−^_t−2_	ΔlnVol^−^_t−3_	ΔlnVol^−^_t−4_	ΔlnVol^−^-5
Singapore												
8542	−0.001	−0.006	0.145**	–	–	–	0.037	–	–	–	–	–
8411	0.123**	−0.094*	0.002	0.164***	–	–	–	–	–	–	–	–
8517	0.184	−0.312**	–	–	–	–	−0.076	−0.002	−0.109	−0.202**	–	–
8486	−0.121*	–	–	–	–	–	–	–	–	–	–	–
8471	0.025	−0.598***	−0.331**	−0.276***	–	–	−0.071	−0.224*	–	–	–	–
**Thailand**												
8471	–	–	–	–	–	–	0.048**	–	–	–	–	–
8703	−0.129*	–	–	–	–	–	–	–	–	–	–	–
8708	–	–	–	–	–	–	0.021	0.077**	–	–	–	–
8542	–	–	–	–	–	–	−0.021	0.010	−0.054	0.112**	0.133***	–
8704	–	–	–	–	–	–	–	–	–	–	–	–
**Malaysia**												
8703	−0.317**	−0.270*	0.488*	–	–	–	0,318	0.429*	−0.495**	0,375	–	–
8541	−0.016	−0.054***	−0.046**	0.001	−0.019	−0.051**	–	–	–	–	–	–
8471	–	–	–	–	–	–	0.197	0.165	0.387**	−0.112	0.244	0.343**
8523	–	–	–	–	–	–	–	–	–	–	–	–
4015	−0.036***	–	–	–	–	–	0.009	–	–	–	–	–
**Indonesia**												
8703	−0.069***	–	–	–	–	–	–	–	–	–	–	–
7219	0.007	0.216	0,022	−1.131***	–	–	–	–	–	–	–	–
3823	–	–	–	–	–	–	–	–	–	–	–	–
6403	0.014	0.067**	–	–	–	–	–	–	–	–	–	–
4802	−0.146***	0.093	0.193***	–	–	–	−0.039	0.172***	–	–	–	–
**Philippines**												
8542	–	–	–	–	–	–	−0.960*	–	–	–	–	–
8471	–	–	–	–	–	–	–	–	–	–	–	–
8543	–	–	–	–	–	–	–	–	–	–	–	–
8544	–	–	–	–	–	–	–	–	–	–	–	–
8443	–	–	–	–	–	–	−10.292***	–	–	–	–	–

Source: Authors' processed data.

*** symbolizes a 1% significance level, ** symbolizes a 5% significance level, and * symbolizes a 10% significance level.

**Table 8:** Long-term NARDL estimate.

Code	C	lnIPI	lnRER	lnVol^-^	lnVol^+^
**Singapore**					
8542	3.686***	−0.458	4.351**	−0.006	0.065
8411	−2.998	3.624**	2.650**	−0.028	0.066
8517	0.230	2.691**	1.789	0.115	0.100
8486	5.044	−1.781	−4.244**	−0.097	−0.035
8471	5.484***	1.057***	−0.209	0.457**	0.426**
**Thailand**					
8471	1.640	4.279	−8.449*	−0.833*	−0.799*
8703	0.526	2.373	−0.443	−0,032	−0.013
8708	2.649***	0.741	0.532	−0.019	−0.050
8542	4.954***	−2.144	1.485	−0.384*	−0.396*
8704	4.158	−1.027	2.308***	−0.035	−0.056
**Malaysia**					
8703	5.392**	−1.279	−0.895	−0.135	−0.256
8541	4.837**	−0.769	−6.268***	−0.030	0.079
8471	2.969***	−0.428	−0.532	0.094	−0.041
8523	1.862	0.333	4.422	−1.488	−1.716*
4015	3.232***	−1.151	−0.728	−0.160**	−0.133*
**Indonesia**					
8703	0.756	0.570	0.857	−0.052	−0.086*
7219	−13.791	3.400	5.490	0.640	0.585
3823	−3.652	−0.778	2.699	−0.194	−0.367
6403	14.888***	0.771	−1.795***	0.021	0.053
4802	5.631***	−0.785	−0.426	−0.450***	−0.488***
**Philippines**					
8542	16.560***	−7.101***	−1.172	−0.364**	0.232
8471	−1.507	0.793	3.092***	0.340**	0.178
8543	−51.194***	21.615	30.883***	0.763	1.704
8544	1.756	0.525	1.094**	−0.048	0.199**
8443	−86.854***	37.752***	7.502	0.096	−0.885

Source: Authors' processed data.

*** symbolizes a 1% significance level, ** symbolizes a 5% significance level, and * symbolizes a 10% significance level.

In the short run, Thailand's export of motor cars and other motor vehicles (8703) decreases with an increase in the volatility of the exchange rate. While a decrease in volatility, increases Thailand's export of automatic data-processing machines (8471), parts and accessories for tractors, motor vehicles (8708), and (8542) electronic integrated circuits. Our finding further revealed that, in the long run, positive and negative change in exchange rate volatility reduces Thailand's export of automatic data-processing machines (8471) and electronic integrated circuits (8542) respectively. The results from the asymmetric analysis revealed that as traders are risk-averse Thailand's export decreases with both positive and negative change in the volatility of the exchange rate.

In the short run, for Malaysia increase in volatility reduces the export of diodes, transistors and similar semiconductor devices (8541) and articles of apparel and clothing accessories (4015). While at different lags increase in volatility decreases and increases the export of motor cars and other motor vehicles (8542) respectively. A negative change in volatility has been found to assert a positive and negative impact on Malaysia's export of diodes, transistors and similar semiconductor devices (8541) at different lags, and increases export of automatic data-processing machines (8471). In the long run, positive and negative change in volatility reduces the exports of discs, tapes, solid-state non-volatile storage devices (8523) and articles of apparel and clothing accessories (4015) respectively. In the case of Malaysia, when the asymmetric effect is employed the effect of volatility on export is more pronounced with more significant coefficients.

The short-run effect revealed that Indonesia's exports of motor cars and other motor vehicles (8703) and flat-rolled products of stainless steel (7219) were negatively affected by the positive change in volatility, while export of footwear with outer soles of rubber, plastics, leather (6403) is positively affected by an increase in volatility. The export of uncoated paper and paperboard (4802) is affected positively and negatively at different by the increase in volatility. The finding further indicates that the negative change in volatility only positively affects uncoated paper and paperboard (4802) export from Indonesia. In the long run, Indonesia's exports of motor cars and other motor vehicles (8703) and uncoated paper and paperboard (4802) decrease with both positive and negative changes in volatility. These findings indicate that exchange rate volatility asserts an asymmetric effect on Indonesia's exports and that Indonesia's export is more susceptible to positive change in volatility in both the short run and long run.

As for the Philippines, in the short run, only electronic integrated circuits (8542) and printing machinery used for printing by means of plates, cylinders and other printing components (8443) are significantly affected by the negative change in volatility. In the long run, an increase in volatility promote the export of insulated, wire, and cable (8544). However, a decrease in volatility promotes the export of automatic data-processing machines (8471) and reduces electronic integrated circuits (8542) export respectively.

From the foregoing discussion, using the nonlinear ARDL approach the exchange rate volatility is broken down into the positive change in volatility (i.e. increase in volatility) and negative change in volatility (i.e. decrease in volatility) to determine the short-run and long-run asymmetric effects of exchange rate volatility. The findings demonstrate that a surge and decline in the volatility of the exchange rate asserts a positive and negative influence on exports in both the short and long term. Different effects (negative and positive) of exchange rate volatility, in all the nonlinear and linear ARDL, can occur due to the risk-averse and risk-taking behaviour of exporters of manufacturing industrial commodities to several trading partners [7,49]. This can cause a different reaction when there is an increase or decrease in the volatility of the exchange rate. Moreover, the different influences of the volatility of the exchange rate may be caused by different volatility sources which can come from micro-structural shocks, exchange rate fundamental behaviour, and the process of signalling future policy innovations [50].

Of the analyzed 5 ASEAN countries, a negative change in volatility has been found to positively affect exports of some commodities. This indicates that with the decline in the volatility of the exchange rate, exporters in the ASEAN-5 will tend to increase their exports. Moreover, a positive change in volatility negatively affects exports as exporters tend to reduce their exports. This also suggests that the exporters are risk-averse in the long term. Findings also confirm that the impact of an increase in volatility is significantly different from a decrease in volatility for most commodities in the long run. The decline in volatility in the ASEAN-5 countries had a greater impact than an increase in volatility. This result is confirmed by the higher number of coefficients whose value is larger in negative volatility. This supports the long-term asymmetric impact of volatility. Therefore, short-term asymmetric adjustments are formed in exchange rate volatility through some different lags between negative and positive volatility in the model, as well as the size and sign of each different lag in a model. Some commodities in the short term, are negatively and positively impacted by the occurrence of the volatility of the exchange rate at different lags. These commodities include commodities 8542 (Electronic integrated circuits) and 8411 (Turbojets, turbo propellers). This indicates that the manufacturing commodities related to high technology can have different effects when adjusting at different lags. This statement is getting stronger because of the consistency of the effects obtained by commodities, both in the linear and nonlinear ARDL. These findings insinuate that maintaining a stable currency in the ASEAN-5 is important for promoting export because any volatile exchange rate will in the long term reduce exports.

The results of the nonlinear ARDL diagnostic test in Appendix B show that all models have negative and significant *ECM*_*t-1*_ coefficients at the 1% and 5% levels of significance. The largest *ECM*_*t-1*_ coefficient (i.e. −0.476) in Singapore is in commodity 8517. This means that the speed of adjustment to long-term equilibrium occurs within 2.1 months. The largest *ECM*_*t-1*_ coefficient (i.e. −0.340) in Thailand is in commodity 8708 with the speed of adjustment to the long-term equilibrium of 2.9 months. Furthermore, the largest *ECM*_*t-1*_ coefficient (i.e. −0.225) in Malaysia is in commodity 8471 with the adjustment speed to the long-term equilibrium of 4.4 months. The largest *ECM*_*t-1*_ coefficient (i.e. −0.651) in Indonesia is in commodity 6403 with the adjustment process to the long-term equilibrium of 1.5 months. The largest *ECM*_*t-1*_ coefficient (i.e. −0.508) in the Philippines with the speed of adjustment to long-term equilibrium for 2 months for commodity 8544.

The Lagrange Multiplier test shows that in almost all models there are no autocorrelation problems. This is evidenced by the p-value which appeared greater than 5%. Likewise, from the probability value i.e. p-value >0.05, the Breusch Pagan heteroscedasticity test shows that in almost all models there is no heteroscedasticity problem. In almost all models the adjusted R^2^ shows that the explanatory variables explained over 50% of the variation in the dependent variables. While the results of the Ramsey RESET test show that in almost all models there is no specification problem. This is because, in almost all models, the probability value is larger than 0.05 the required level of significance.

To test the cumulative short and long-term asymmetric effect, the Wald test is conducted and findings indicate that in the short run, there exists an asymmetric impact of volatility on exports for one commodity (8411) in Singapore, two commodities (8471, 8704) in Thailand, two commodities (8541, 8471) in Malaysia, and four commodities (8542, 7219, 6403, 4802) in Indonesia. While in the long-term the result indicates that, in almost all models, the long-term asymmetric effect of volatility on exports exists except for commodity codes 8517 (Singapore); 8542 (Thailand); 4015 (Malaysia); 7219 (Indonesia); 8543, and 8443 (Philippines).

### 4.5 Quantile regression

For a more robustness check of the analytical findings, quantile regression is also estimated. Tables 9–13 report the results of the quantile regression estimate of exchange rate volatility for the ASEAN-5 countries. Tables 9 and 10 display that the impact of volatility in ASEAN-5 varies substantially with a robust influence on export at higher quantiles (90 percentile) in Singapore and Thailand, and a lower effect at lower quantiles (10 percentile). In Malaysia, the volatility of the exchange rate asserts a strong impact on export at medium quantiles (50 percentile) and a lower effect at higher quantiles (90 percentile). This indicates that the volatility of the exchange rate weakly and significantly influences export in Malaysia. Exchange rate volatility in Indonesia has a robust influence on export at lesser quantiles of 10 and 25 percentiles. These findings reveal that the impact is much higher at lesser quantiles although the strongest effect appeared at the medium quantiles. This indicates that the volatility of the exchange rate significantly affects export. In the case of the Philippines, the effect of exchange rate volatility appeared at lesser quantiles of 10 and 25 percentiles. This finding proves that the impact is much higher at lesser quantiles, and volatility does not influence export significantly. Therefore, from the quantile regression results, the study concludes that in 4 out of 5 ASEAN-5 countries, exchange rate volatility asserts a significant and strong influence on export based on the consistency of the empirical estimates.

**Table 9:** Results of Singapore (8411) quantile regression.

	Quantile	Coefficient	Std. Error	t-Statistic	Prob.
lnIPI_SG_8411	0.100	3.830850	1.274912	3.004795	0.0032
	0.250	4.142016	1.597376	2.593013	0.0106
	0.500	16.08744	1.889058	8.516115	0.0000
	0.750	18.45642	1.446297	12.76115	0.0000
	0.900	17.35889	1.964061	8.838267	0.0000
lnRER_SG_8411	0.100	−1.953733	0.907927	−2.151862	0.0332
	0.250	−0.269687	1.223478	−0.220426	0.8259
	0.500	5.964999	0.882595	6.758476	0.0000
	0.750	6.258852	0.859505	7.281928	0.0000
	0.900	6.349625	1.124755	5.645340	0.0000
lnVOL_SG_8411	0.100	−0.505892	0.142556	−3.548716	0.0005
	0.250	−0.659280	0.186580	−3.533487	0.0006
	0.500	−0.504354	0.283436	−1.779424	0.0775
	0.750	−0.317315	0.237290	−1.337247	0.1834
	0.900	−0.507203	0.279798	−1.812747	0.0721
C	0.100	−6.682634	5.963717	−1.120549	0.2645
	0.250	−8.175641	7.512255	−1.088307	0.2784
	0.500	−64.84795	9.085462	−7.137551	0.0000
	0.750	−75.82892	6.981812	−10.86092	0.0000
	0.900	−70.30316	9.479125	−7.416630	0.0000

Source: Authors' processed data.

**Table 10:** Results of Thailand (8542) quantile regression.

	Quantile	Coefficient	Std. Error	t-Statistic	Prob.
lnIPI_THAI_8542	0.100	0.385828	0.289394	1.333225	0.1848
	0.250	0.472788	0.360915	1.309971	0.1925
	0.500	0.240062	0.814307	0.294805	0.7686
	0.750	−0.114728	0.679128	−0.168934	0.8661
	0.900	−0.368130	0.560659	−0.656603	0.5126
lnRER_-THAI_8542	0.100	−1.296766	0.303074	−4.278715	0.0000
	0.250	−0.714832	0.373566	−1.913537	0.0578
	0.500	0.814275	0.489656	1.662953	0.0987
	0.750	1.750918	0.219373	7.981467	0.0000
	0.900	1.927306	0.167108	11.53327	0.0000
lnVOL_THAI_8542	0.100	−0.079029	0.057343	−1.378185	0.1705
	0.250	−0.139852	0.062102	−2.251968	0.0260
	0.500	−0.138327	0.050431	−2.742875	0.0069
	0.750	−0.077031	0.036636	−2.102577	0.0374
	0.900	−0.065604	0.030711	−2.136165	0.0345
C	0.100	8.121855	1.400221	5.800409	0.0000
	0.250	8.551641	1.741081	4.911686	0.0000
	0.500	11.67902	3.767106	3.100262	0.0024
	0.750	14.61365	3.087597	4.733016	0.0000
	0.900	16.07395	2.547388	6.309971	0.0000

Source: Authors' processed data.

**Table 11:** Results of Malaysia (8542) quantile regression.

	Quantile	Coefficient	Std. Error	t-Statistic	Prob.
lnIPI_MLY_8542	0.100	2.245154	0.787292	2.851743	0.0050
	0.250	1.637305	0.535872	3.055404	0.0027
	0.500	1.003604	0.688478	1.457713	0.1473
	0.750	1.257320	0.810084	1.552086	0.1230
	0.900	0.800877	0.855740	0.935888	0.3510
lnRER_MLY_8542	0.100	1.138139	0.473439	2.403980	0.0176
	0.250	0.772999	0.289768	2.667650	0.0086
	0.500	1.067839	0.347489	3.073018	0.0026
	0.750	2.080979	0.387241	5.373865	0.0000
	0.900	2.279855	0.308115	7.399375	0.0000
lnVOL_MLY_8542	0.100	−0.192885	0.098268	−1.962844	0.0518
	0.250	−0.129201	0.068505	−1.886015	0.0615
	0.500	−0.197127	0.087387	−2.255793	0.0257
	0.750	−0.136476	0.121197	−1.126060	0.2622
	0.900	−0.078560	0.125053	−0.628212	0.5310
C	0.100	2.222083	3.571924	0.622097	0.5350
	0.250	5.211684	2.407268	2.164979	0.0322
	0.500	8.196718	3.100497	2.643679	0.0092
	0.750	8.322100	3.570446	2.330829	0.0213
	0.900	10.99066	3.799463	2.892688	0.0045

Source: Authors' processed data.

**Table 12:** Results of Indonesia (6403) quantile regression.

	Quantile	Coefficient	Std. Error	t-Statistic	Prob.
lnIPI_INA_6403	0.100	−1.253845	0.326839	−3.836281	0.0002
	0.250	−2.191431	0.499335	−4.388697	0.0000
	0.500	−1.308712	2.816711	−0.464624	0.6430
	0.750	−0.957280	1.987720	−0.481597	0.6309
	0.900	−0.844453	3.303275	−0.255641	0.7986
lnRER_INA_6403	0.100	−0.650022	0.550522	−1.180737	0.2398
	0.250	0.915692	0.430688	2.126116	0.0354
	0.500	1.374391	0.771952	1.780411	0.0773
	0.750	1.878259	0.494180	3.800756	0.0002
	0.900	1.872924	0.713471	2.625089	0.0097
lnVOL_INA_6403	0.100	−0.243963	0.057304	−4.257365	0.0000
	0.250	−0.325756	0.036717	−8.872026	0.0000
	0.500	−0.225985	0.054605	−4.138516	0.0001
	0.750	−0.208760	0.053445	−3.906040	0.0001
	0.900	−0.153844	0.051509	−2.986763	0.0034
C	0.100	24.51319	4.219917	5.808929	0.0000
	0.250	15.51770	3.055769	5.078166	0.0000
	0.500	6.377936	8.186797	0.779051	0.4373
	0.750	0.129400	6.025116	0.021477	0.9829
	0.900	−0.773434	9.661080	−0.080057	0.9363

Source: Authors' processed data.

**Table 13:** Results of Philippines (8542) quantile regression.

	Quantile	Coefficient	Std. Error	t-Statistic	Prob.
lnIPI_PHIL_8542	0.100	−2.499476	2.183467	−1.144728	0.2544
	0.250	−2.022127	1.759601	−1.149197	0.2526
	0.500	4.617505	1.471622	3.137698	0.0021
	0.750	6.394326	1.416399	4.514495	0.0000
	0.900	4.673304	3.624178	1.289480	0.1995
lnRER_PHIL_8542	0.100	−4.049071	2.043734	−1.981212	0.0496
	0.250	−3.640286	1.779483	−2.045699	0.0428
	0.500	−1.194154	0.817805	−1.460194	0.1466
	0.750	−0.791214	0.717080	−1.103382	0.2719
	0.900	−0.002781	1.237544	−0.002247	0.9982
lnVOL_PHIL_8542	0.100	−1.037109	0.166282	−6.237039	0.0000
	0.250	−0.755418	0.172461	−4.380218	0.0000
	0.500	−0.259151	0.172922	−1.498662	0.1363
	0.750	0.036568	0.116457	0.314006	0.7540
	0.900	−0.014207	0.123464	−0.115069	0.9086
C	0.100	41.63892	16.64810	2.501121	0.0136
	0.250	36.94536	13.65949	2.704740	0.0077
	0.500	−4.889057	9.426329	−0.518660	0.6049
	0.750	−15.67002	7.087935	−2.210802	0.0288
	0.900	−10.32068	13.51767	−0.763496	0.4465
					
					

Source: Authors' processed data.

## 5 Conclusions

In this study, we analyzed the influence of the volatility of the exchange rate on the exports of main industrial manufactured goods in ASEAN-5 countries. Using both the symmetric and asymmetric modelling approach to monthly export data for the period January 2007 and March 2019, findings indicate that volatility of the exchange rate asserts a significant influence on the exports of 13 commodities in the short run. However, finding from the nonlinear ARDL modelling approach revealed that the volatility asserts a significant impact on more commodities as 19 commodities were significantly affected.

In the short term, volatility asserts a positive and significant influence on commodities (8542, 8411) in Singapore, (8471, 8708, 8542) in Thailand, (8471) in Malaysia, (6403) in Indonesia, (8544) in the Philippines. The adverse effect of volatility on exports appeared in (8517) commodity in Singapore, (8704) commodity in Thailand, (8542) commodity in Malaysia, and (7219) commodity in Indonesia. In addition, volatility also asserts a negative and positive influence on different lags in the Philippines (8542). In the long run, the volatility asserts a significant positive influence on the Philippines (8471) and a significant negative influence on Thailand (8704, 8703, 8708, 8542), Malaysia (8523), Indonesia (8703), Philippines (8542, 8544). Moreover, in the short run, the strategy from the nonlinear ARDL revealed that a decrease in exchange rate volatility asserts a significant positive impact on (8471, 8708, 8542) commodities in Thailand, (8471) commodity in Malaysia, (4802) commodity in Indonesia and negatively affect (8517, 8471) commodities in Singapore, (8542, 8443) commodities in the Philippines. Additionally, a reduction in volatility asserts both negative and positive impacts on export at different lags in Malaysia (8542). On the other hand, in the short term, a rise in volatility asserts a positive and significant impact on the export of; (8542) commodity in Singapore, (6403) commodity in Indonesia, and a negative effect on (8517, 8486, 8471) commodities in Singapore, (8703) commodity in Thailand, (8541, 4015) commodities in Malaysia, (8703, 7219) commodities in Indonesia. Furthermore, at different lags, an increase in exchange rate volatility asserts both negative and positive impacts on export for (8411) commodity in Singapore.

In the long term, a decrease in volatility asserts a positive and significant positive impact on Singapore (8471) commodity, Philippines (8471) commodity and a significant negative effect on Thailand (8471 and 8542) commodities, Malaysia (4015) commodity, Indonesia (4802) commodity, and the Philippines (8542) commodity. However, an increase in volatility asserts a positive and significant influence on Singapore (8471) commodity, the Philippines (8544) commodity, and a significant negative effect on Thailand (8471 and 8542) commodities, Malaysia (8523 and 4015) commodities, Indonesia (8703 and 4802) commodities. Findings also indicate that a decrease in exchange rate volatility has a greater impact than an increase in volatility in ASEAN-5.

In line with the analytical findings, we, therefore, acclaim the need for government to give emphasis and fast-track both the negative and positive changes in the volatility of the exchange rate. Since there exists an asymmetric effect, keeping the exchange rate stable would be very crucial in the ASEAN-5 countries because of the exporters’ risk-averse behaviour. To maintain the stability of the exchange rate, the government needs to maintain sufficient foreign exchange reserves and increase the level of domestic investment. Additionally, business players need to differentiate and improve the competitiveness of their commodity exports. Product differentiation can be realized by innovating and adopting new and efficient technology.

### 5.1 Limitation

The main constraint of this research work lies in its focus on only a few commodities exported by the ASEAN-5 countries to their major trading partners while investigating the influence of exchange rate volatility on manufacturing exports. Furthermore, apart from, the main variable of interest, the study only controlled for the industrial production index (IPI). To augment this study, future work needs to explore more about the exchange rate volatility and manufacturing exports in other countries for which data are available for many exportable commodities and also control for other key determinants of exports.

## Author contribution statement

Rossanto Dwi Handoyo, Ph.D.; Sesotya Sesotya; Kabiru Hannafi Ibrahim, Ph.D; Tamat Sarmidi, Ph.D.; Tri Haryanto, Ph.D.: Conceived and designed the experiments; Performed the experiments; Analyzed and interpreted the data; Contributed reagents, materials, analysis tools or data; Wrote the paper.

## Funding statement

This research did not receive any specific grant from funding agencies in the public, commercial, or not-for-profit sectors.

## Data availability statement

The data is available upon request.

## Declaration of interest's statement

The authors declare no competing interests.

## Figures and Tables

**Table:** 

Code	ECM_t-1_	LM (Prob)	Hetero (Prob)	Adj. R^2^	RESET (Prob)
Singapore					
8542	−0.156***	0.867	0.706	0.815	0.387
8411	−0.042***	0.387	0.989	0.905	0.7472
8517	−0.477***	0.686	0.000	0.359	0.6176
8486	−0.092**	0.502	0.039	0.845	0.6636
8471	−0.579***	0.795	0.766	0.388	0.801
**Thailand**					
8471	−0.060**	0.213	0.450	0.767	0.7405
8703	−0.258***	0.422	0.169	0.557	0.1790
8708	−0.195***	0.412	0.460	0.803	0.6343
8542	−0.226***	0.224	0.537	0.787	0.2145
8704	−0.337***	0.156	0.126	0.600	0.1986
**Malaysia**					
8703	−0.143**	0.455	0.184	0.714	0.4227
8541	−0.028**	0.166	0.266	0.902	0.2326
8471	−0.143***	0.887	0.125	0.793	0.4397
8523	−0.137***	0.965	0.197	0.861	0.3577
4015	−0.089***	0.014	0.982	0.874	0.7566
**Indonesia**					
8703	−0.307***	0.718	0.325	0.884	0.2709
7219	−0.257***	0.367	0.207	0.484	0.0013
3823	−0.150***	0.998	0.000	0.758	0.3537
6403	−0.109**	0.045	0.527	0.799	0.1748
4802	−0.250***	0.764	0.057	0.536	0.5046
**Philippines**					
8542	−0.251***	0.939	0.000	0.903	0.4683
8471	−0.306***	0.774	0.376	0.789	0.0397
8543	−0.214***	0.043	0.610	0.735	0.2076
8544	−0.391***	0.361	0.899	0.765	0.541
8443	−0.340***	0.735	0.712	0.692	0.9185

Source: Authors' processed data.

*** symbolizes a 1% significance level, ** symbolizes a 5% significance level, and * symbolizes a 10% significance level.

**Table:** 

Code	ECM_t-1_	LM (Prob)	Hetero (Prob)	Adj. R^2^	RESET (Prob)	Wald (Prob)
Singapore						
8542	−0.195***	0.958	0.676	0.819	0.536	0.038
8411	−0.363***	0.463	0.216	0.915	0.011	0.000
8517	−0.476***	0.749	0.004	0.359	0.950	0.465
8486	−0.268***	0.079	0.101	0.852	0.299	0.010
8471	−1.053***	0.969	0.347	0.451	0.204	0.000
**Thailand**						
8471	−0.157***	0.329	0.149	0.760	0.919	0.024
8703	−0.279***	0.494	0.143	0.546	0.404	0.011
8708	−0.340***	0.382	0.556	0.806	0.953	0.003
8542	−0.210***	0.286	0.392	0.327	0.296	0.612
8704	−0.501***	0.909	0.117	0.622	0.033	0.000
**Malaysia**						
8542	−0.302***	0.406	0.180	0.743	0.567	0.008
8541	−0.222***	0.186	0.858	0.916	0.714	0.000
8471	−0.225***	0.845	0.271	0.807	0.510	0.041
8523	−0.166***	0.856	0.331	0.858	0.574	0.060
4015	−0.184***	0.007	0.613	0.879	0.314	0.183
**Indonesia**						
8703	−0.441***	0.337	0.189	0.892	0.682	0.000
7219	−0.256***	0.318	0.133	0.491	0.002	0.199
3823	−0.355***	0.425	0.000	0.771	0.360	0.002
6403	−0.651***	0.783	0.321	0.828	0.996	0.000
4802	−0.353***	0.392	0.299	0.602	0.519	0.001
**Philippines**						
8542	−0.352***	0.668	0.000	0.909	0.200	0.000
8471	−0.408***	0.457	0.124	0.791	0.247	0.038
8543	−0.239***	0.039	0.665	0.725	0.045	0.236
8544	−0.508***	0.035	0.761	0.788	0.013	0.000
8443	−0.434***	0.755	0.757	0.714	0.690	0.741

Source: Authors' processed data.

*** symbolizes a 1% significance level, ** symbolize a 5% significance level, and * symbolizes a 10% significance level.
